# Results and insights from the NCSU Insect Museum GigaPan project

**DOI:** 10.3897/zookeys.209.3083

**Published:** 2012-07-20

**Authors:** Matthew A. Bertone, Robert L. Blinn, Tanner M. StanfieldKelly J. Dew1, Katja C. Seltmann, Andrew R. Deans

**Affiliations:** 1 Department of Entomology, North Carolina State University, Campus Box 7613, Raleigh, NC, 27695-7613, USA; 2 American Museum of Natural History, Central Park West at 79th St., New York, NY 10024-5192 USA

**Keywords:** panorama, gigapixel, megapixel, specimen, collection, imaging system

## Abstract

Pinned insect specimens stored in museum collections are a fragile and valuable resource for entomological research. As such, they are usually kept away from viewing by the public and hard to access by experts. Here we present a method for mass imaging insect specimens, using GigaPan technology to achieve highly explorable, many-megapixel panoramas of insect museum drawers. We discuss the advantages and limitations of the system, and describe future avenues of collections research using this technology.

## Introduction

Insect specimens are integral to basic entomological research such as systematics, ecology, and applied sciences. However, most are preserved dried on pins and stored in large collections, where they remain difficult to physically access (e.g., requiring permissions and/or expensive travel). This situation leads to a massive underutilization of specimens and their associated data. While the process of physically sending (i.e., loaning) materials alleviates the need to travel to collections, it is time consuming for collection managers and difficult for the borrower to specify which individuals are needed without knowledge of the true holdings (e.g., requesting from series of undetermined specimens). More importantly, whenever specimens are removed from their drawers they are at risk of being exposed to unfavorable conditions, including handling by untrained users, losses during transit or being misplaced, and insufficient temporary curatorial practices.

It is essential for insect collections to have a web presence and disseminate information online. Online databases of public and private collections are common practice, and usually include specimen names and taxonomic status, number of individuals of each taxon, and data from labels (such as localities, dates and other information regarding the specimen’s provenance). Some collections even host images of their materials, though it is usually limited to a few photographs of exemplars or valuable specimens (e.g., types). Despite these advances, very few avenues exist to thoroughly browse the holdings of any one collection, visually, and to evaluate the extent/quality of its specimens and the degree to which they are curated.

GigaPan (www.gigapan.com ) was initially developed through a collaboration between Carnegie Mellon University and the NASA Ames Intelligent Robotics Group for use on NASA’s Mars Rovers (Spirit and Opportunity). It has since become a commercially available hardware and software, used to achieve many-megapixel to gigapixel (i.e., billions of pixels) images that are then represented as highly-navigable panoramas. The basic product consists of a robot that can be fitted with any digital camera (depending on camera and robot model) and mounted on typical tripod threads. Once initiated, the robot positions the camera to frame individual images across a designated area of interest and uses a robotic “finger” (or remote release) to engage the camera, which captures multiple, overlapping tiles (i.e., photos). GigaPan software is then used to stitch the resulting photos into one large panorama that has a maximum resolution roughly matching the resolution of each individual image, but across a much larger area. Further, panoramas currently can be hosted on the GigaPan website where viewers may add general comments and take snapshots of specific areas, either with annotations describing the importance of the area or questions about it. Though commonly used for capturing vast landscapes and large events, the potential of these panoramas is far reaching.

With about 1.5 million specimens, the North Carolina State University Insect Museum (http://insectmuseum.org ) is the largest insect collection in North Carolina, and among the largest in the southeastern United States. The pinned collection is strong in several groups, including Hemiptera (bugs, especially Auchenorrhyncha, the holdings of which are world-renowned), Anthophila (bees, especially Megachilidae), and Pyralidae (snout moths). At a moderate size, the NCSU Insect Museum presents an important, but manageable, resource for understanding modern digitization potential of insect collections. Here we present results and insights gained from our efforts to image whole drawers using GigaPan technology. We provide details on how to achieve similar results, describe the advantages and drawbacks of the system, and discuss outcomes of the project.

## Methods

### Existing infrastructure

The NCSU Insect Museum has roughly 2,700 insect drawers in use, stored in 184 12- or 24-drawer metal cabinets. Drawers are U.S. National Museum (USNM) style, with the following dimensions: 45.72cm W × 45.72cm D × 7.3cm H (18"W × 18"D × 2–7/8"H; outer measurements) and 41.28cm W × 42.55cm D × 5.87cm H (16–1/4"W × 16–3/4"D × 2–5/16"H; inner measurements).

### Equipment

**Figure 1.**
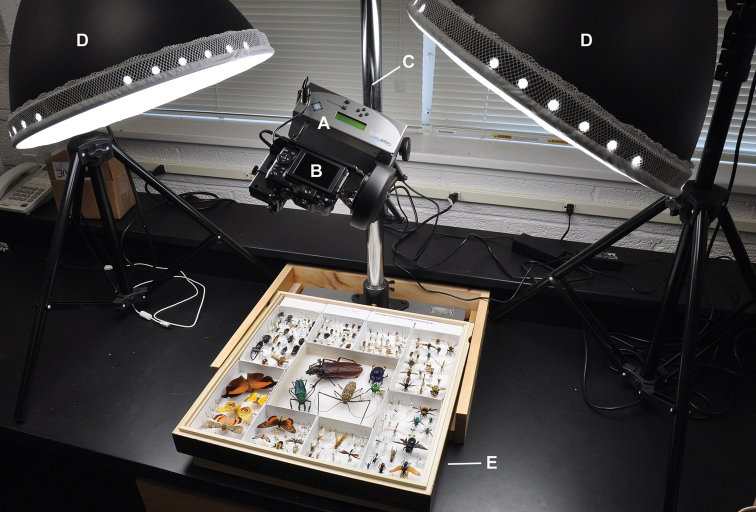
The complete imaging station. **A** GigaPan Epic 100 (“silver model”) robot **B** Canon PowerShot G11 camera **C** copy stand **D** light with continuous compact fluorescent bulbs **E** insect drawer.

We employed a GigaPan EPIC 100 (“silver model”), oriented horizontally on a copy stand and paired with a Canon PowerShot G11 camera. We retrofitted the GigaPan with an A/C adapter (Sargent et al. 2010) and bought a commercial A/C adapter for the Canon to alleviate the need for disposable batteries and/or charging requirements. Our lighting needs were satisfied by dual Interfit Super Cool-Lite 9 lights, each with nine 28W compact-fluorescent bulbs that produce continuous daylight spectrum (5000–5500K). Both lights were equipped with the included diffusion covers for softer lighting. Other diffused lights delivering this spectrum would be suitable. Most of the stitching was performed on an Intel i7 quad core Apple iMac (2.8 GHz, 4,096 GB RAM). The complete imaging station (without the computer) is illustrated in Figure 1.

### Settings

Camera settings were based largely on those described in the GigaPan tutorials (http://gigapan.org/cms/videos ) and manual (Gigapan Systems 2010), with the white balance set to daylight fluorescent (best balance for the lighting described above) and the field of view (FOV) for the camera set to 11.5º on the GigaPan unit. The FOV is dependent on the camera model, so this number is specific to the Canon PowerShot G11. The aperture was set to f/8.0 (the smallest available for the camera) to achieve the greatest depth of field (DOF; 3.5cm). The distance of the GigaPan robot plus camera was set to about 46.35 cm (18.25”) from the base of the copy stand [about 43.2cm (17”) above the average pinned specimen]. This height is beneficial for optimizing the DOF, quality, and size of the images at full optical zoom, while reducing curvature (see Results) and keeping the number of photos (~35 per drawer) manageable with respect to time and storage capabilities. All images were shot as large, super-fine quality JPEGs (3,648 × 2,736 pixels). The focus was locked to prevent the variable amount of time needed for the auto focus, which could result in the camera not completing the process before the robot moves to the next position. A custom timer delay of 2 sec was also added to ensure the unit was stable during photo capture. In conjunction, the “Time per Pic” on the robot was set to 4.5 sec, so movement would not occur during capture. All settings were saved in one of the two custom settings slots (C1 or C2) available on the Canon G11 for recall when the camera is turned on.

### Imaging workflow

**Figure 2.**
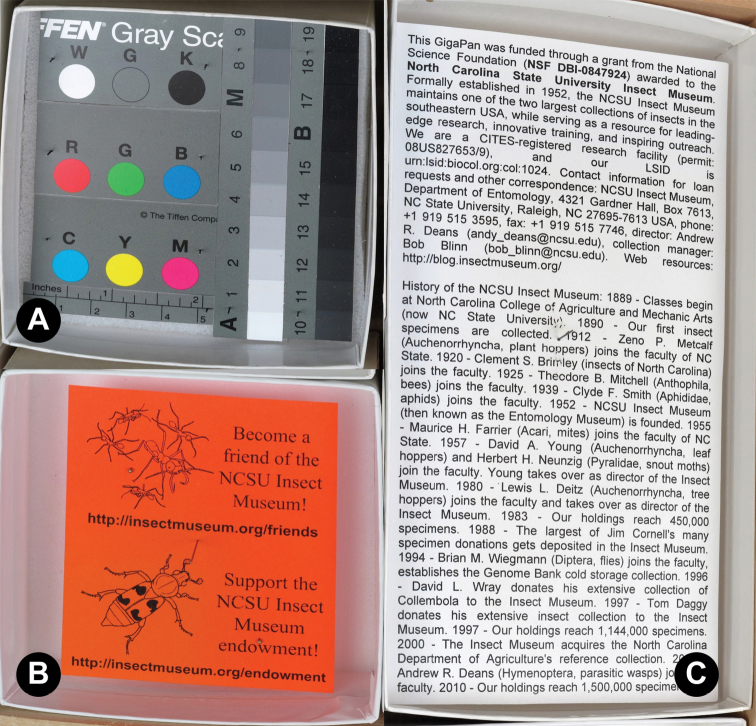
Color standards and white space filler. **A** Kodak Tiffen Color Separation Guide **B, C** text/picture white space filler.

Drawers were placed within the confines of a custom jig on the copy stand, with the lid removed. To prevent white space from interfering with the camera’s ability to focus (an issue sometimes encountered, despite locking the focus), a Kodak Tiffen Color Separation Guide (ASIN: B00009R7G9; trimmed to fit inside a unit tray) and printed matter were placed inside empty unit trays (Fig. 2). Initially, the “New Panorama” process was begun on the GigaPan robot to define the boundaries of the drawer to be captured by the camera, and verify that the camera settings were in place and correct. After the initial setup, the Epic 100 was engaged using the “Last Panorama” function, unless the image area needed to be modified. While the robot and camera were working drawer preparation occurred for the next one in line, reducing the overall amount of time needed. After capturing all images on the camera’s memory card, each completed insect drawer was given a label with the date the panorama was taken and returned to the collection. Photos for each panorama (usually n=35) were delivered manually onto a computer hard drive or external hard drive (through the computer) directly from the camera using a USB cable; using a cable bypassed the need to remove the camera and memory card, potentially moving the unit from its set positions (required for using “Last Panorama” function properly). All photos were checked during/after transfer for errors, especially out of focus images, and reshot if necessary. Stitching was then initiated manually on the computer by opening the drawer images, previously transferred from the camera, in the GigaPan Stitch software (version 1.0.0804; provided by GigaPan); stitching was done either singly or as a batch of multiple drawers (10–20 at a time). Batches were possible by opening any existing .gigapan file in the stitch software and using the “New Gigapan” function (File > New Gigapan) to select the new set of photos to stitch; repeating this process resulted in multiple stitch windows open concurrently on the computer. All panoramas were checked during the preview phase of the stitching to ensure that no errors existed, most frequently misaligned tiles. If a re-stitch did not work the drawer was reshot. Finally, stitched sets were stored locally, backed up by external hard drives, and uploaded to the GigaPan website (either singly or as batches in the same manner as described above for stitching). During uploads, each panorama was given a brief description and several keywords (usually standard words like “insect” and “museum”, the order, and families present in each drawer). Throughout the entire process, custom paperwork was used to record all drawers being imaged and the status of their progression. Also, to ensure that the lights did not overheat a cool-down time of 5–10 minutes was added after shooting about 10–15 panoramas. A schematic of the entire workflow can be seen in Figure 3 and a video tutorial can be found at http://purl.oclc.org/NET/NCSU/gigapanvid .

**Figure 3.**
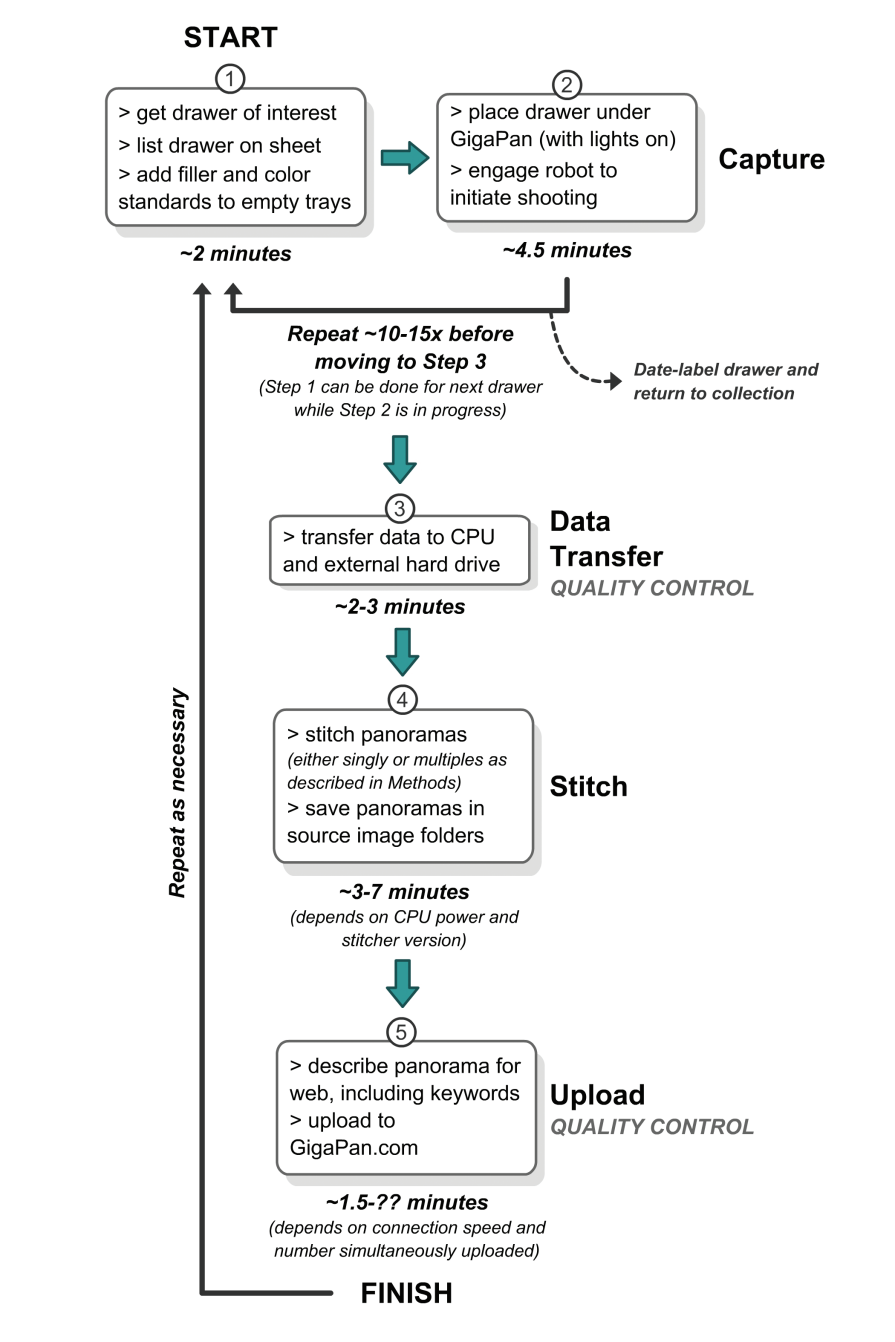
Schematic of project workflow. Note: times are rough estimates and prone to change depending on the efficiency of several steps.

## Results

### General

**Figure 4.**
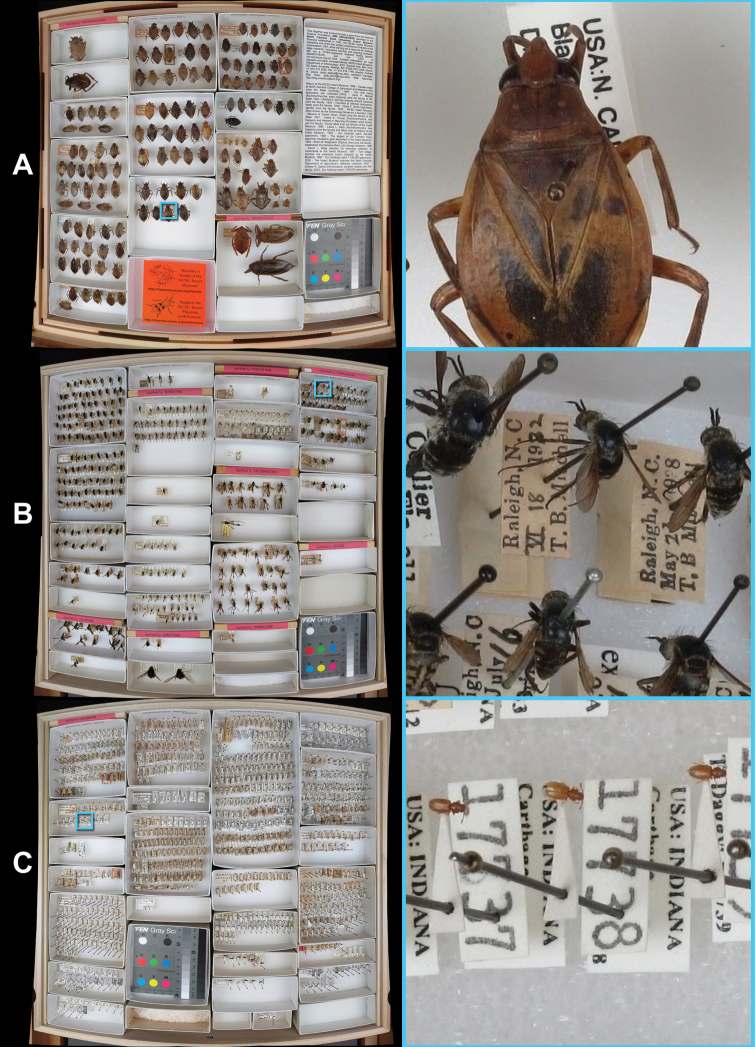
Examples of typical drawers, showing larger specimens, average specimens, and smaller specimens (**A, B & C**, respectively). Left – full drawer image; Right – zoomed to full resolution. **A** Belostomatidae 1 (http://gigapan.org/gigapans/96136 ) **B** Bombyliidae 5 (http://gigapan.org/gigapans/89195 ) **C** Silvanidae 2 (http://gigapan.org/gigapans/95947 )

**Figure 5.**
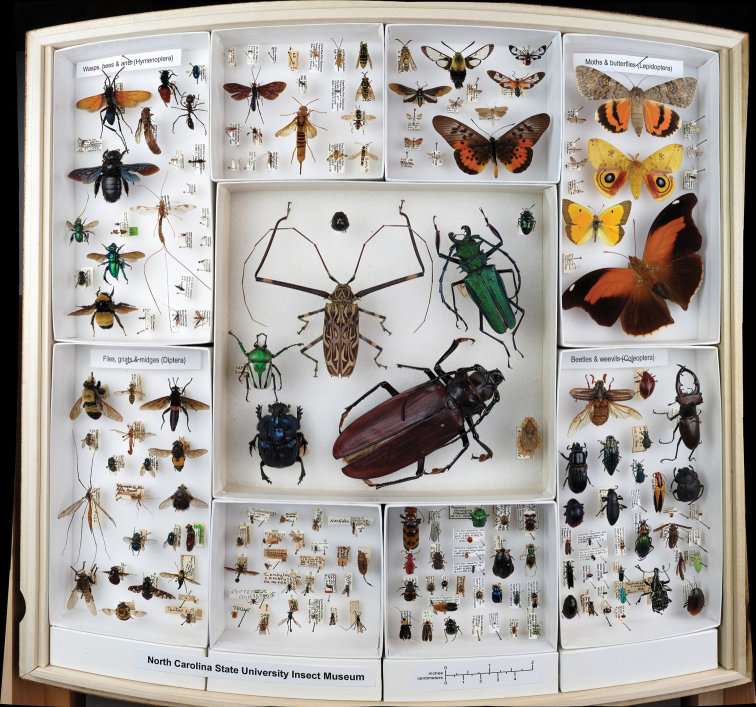
Example of a thematic drawer displaying the diversity of the four largest insect orders (http://gigapan.org/gigapans/49310 ). Clockwise from Top Left: Hymenoptera (wasps, ants & bees), Lepidoptera (moths & butterflies), Coleoptera (beetles), and Diptera (true flies). The drawer also serves as an outreach tool by containing some mistakes for people to identify and further learn the differences between the orders.

As of March 1, 2012, the NCSU Insect Museum had 2,124 panoramas uploaded (http://gigapan.org/profiles/ncsuinsectmuseum ), or about 79% of the ~2,700 drawers. Figure 4 illustrates typical drawers, while Figure 5 shows a specialty drawer that was assembled to show insects by theme (in this case the diversity of the four largest insect orders). Final panoramas averaged about 208 megapixels in size (14,700 × 14,150 pixels).

### Time to drawer completion

Average time for completing a drawer – from inserting color standards and text (not including time needed to initially create space in each drawer) through stitching and uploading – was from 12–50+ minutes. Each step required the following amount of time (single or batch; process further described in Fig. 3): drawer prep and filler placement - ~2 mins; image capture - ~4.5 mins; data transfer - ~1–3 mins (batch of 10–15); stitching images - ~3–14 mins (batch of 10–20); uploading - ~1.5+ mins (batch of 10–20). These figures were generalized over the entire life of the project, and using the latest versions of the stitch/upload software while opening multiple stitch/upload windows (described above in Methods) greatly reduced time needed to create and make public the panoramas; future, faster versions of the software should reduce these times even further. Other variables also exist that affect speed, including CPU processing power and internet connectivity (e.g., wireless vs. hard-wired connection speeds, the former usually resulting in slower uploads). Overall these figures represent a conservative estimate of 25 mins to complete each drawer.

### Data storage requirements

About 150MB (typical range: 140–165MB) of storage space was required for each drawer’s complete panorama data (including original photos, raw tile data, and gigapan panorama file). Thus, for the entire 2,700 drawer collection, ~405 gigabytes of storage space was needed. These figures are based on JPEG images with an average size of 1.8–2.6MB each (resulting from size/resolution settings described in Methods).

### Panorama quality

**Figure 6.**
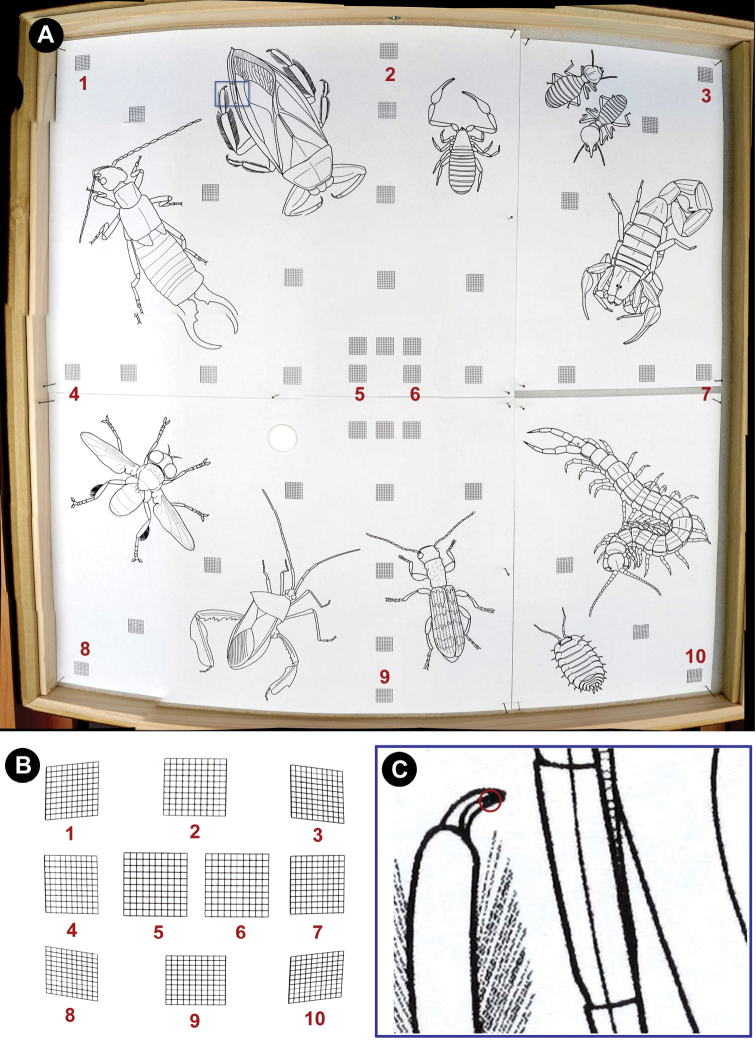
Panorama measures of distortion and resolution. **A** drawer with illustrations and 1cm x 1cm (1mm subunit) grids spanning the panorama **B** comparison of distortion produced across the top (1, 2, & 3), middle (4, 5, & 6), and bottom (7, 8, & 9) of drawer in A **C** smallest resolvable difference between black and white (~80µm) at 1:1 magnification (from blue rectangle in **A**).

Panorama qualities, including resolution and distortion, were measured using a test drawer and the resulting panorama (Fig. 6). As expected, curvature/distortion (see Discussion) was found to be greatest near the edges of the drawers, i.e. furthest from the center. Specifically, there was a 20% reduction of all lengths measured from the corners and sides of the panorama (1, 3, 4, 7, 8, & 10 in Fig. 6B), and a 20% reduction of the vertical measurements at the top and bottom positions (horizontal measurements of top and bottom appear unaffected; 2 & 9 in Fig. 6B). Further, some skewing of measurements occurred, especially at the corners of the panorama, resulting in distorted areas (see 1, 3, 8, & 10 in Fig. 6B). As for resolution, the smallest resolvable structure on a fully-zoomed panorama (discernible white space between two black spaces; Fig. 6C) measured about 80µm; thus structures smaller than this may not be discernible using the current camera optics and settings.

### Online metrics

Panoramas on the NCSU Insect Museum profile at Gigapan.com (n=2,124) have been viewed a total of 326,252 times, at an average of 153.6 views and a median of 94 views. We do not have data on the percentage of unique visitors. The award-winning, specialty drawer “The Big Four” has the most views for a single panorama (24,054 as of the date above), largely resulting from widespread attention gained from GigaPan and media covering the panorama contest during the first meeting of the Fine International Conference on Gigapixel Imagery for Science (http://www.cmu.edu/news/archive/2010/September/sept30_gigapixelshow.shtml ). Eighteen drawers have over 1,000 views, including both special panoramas and typical museum drawers.

## Discussion

This project represents the largest and most complete effort to image and publicly-share an entire insect collection, with over 2,000 drawer panoramas available. The panoramas have been viewed many thousands of times and interactions with both experts and laypeople have occurred. While the project is not yet complete, several outcomes have materialized from the effort.

Unsolicited, remote curation has happened. Word of our insect drawer images spread quickly among insect systematists and we rapidly received communications that enhanced our holdings. In one instance, a taxonomist at a natural history museum in Ottawa, ON (837 miles north of the NCSU Insect Museum) determined a series of froghopper (Hemiptera: Cercopidae) specimens to species from an “unsorted insects” drawer (http://gigapan.org/gigapans/41421/snapshots/120403/ ). Along the same lines, a world bumble bee (Hymenoptera: Apidae: *Bombus*) expert provided a species name for an undetermined specimen (http://gigapan.org/gigapans/49310/snapshots/139687 ), and a lanternfly (Hemiptera: Fulgoridae) expert determined several specimens to species. Further, a velvet ant (Hymenoptera: Mutillidae) specialist identified several specimens (http://gigapan.org/gigapans/60116 ), provided new information on the taxonomic status (synonymies) of several species, and helped resolve the identity of a wasp that had become decoupled from its pin. All interactions were communicated between coordinating members of the museum, and steps were taken to update the collection based on input from the interaction. Additionally, the project has enabled more informed donations: a world expert has contacted us to say she is using our GigaPan images to better understand our current holdings, so that she can then divide up her personal collection between natural history museums more efficiently. She wants to maximize the taxonomic coverage of her donation to our museum.

We also successfully reached out and engaged the public using these panoramas. For example, non-entomologists commented on artistic representation (http://gigapan.org/snapshots/119341/comments ), made humorous comments about the insect specimens (http://gigapan.org/snapshots/117944/comments ), and asked questions about insect biology (http://gigapan.org/snapshots/147239/comments ). The creation and promotion of more thematic drawers, for example teaching concepts using the panoramas (as in Fig. 5) or testing knowledge using Easter eggs and treasure hunts, could easily draw more attention from the public and contribute to our mission for increased outreach, all resulting in added interest in our science.

During the project, several unanticipated outcomes occurred. One was the linking of specimen snapshots to panoramas of their locality/habitat (based on label information). Unsolicited, another member of the GigaPan community and part of the Fine Outreach for Science group, took a panorama of the cloud forest habitat in Costa Rica where one of our leafhopper specimens was collected, and linked it through a snapshot (http://gigapan.org/snapshots/127411/comments ). The practical applications of these data are plentiful, including using the panorama of the habitat to estimate plant diversity related to insect specimens, or change in habitat over time. Researchers could use a GigaPan at their collecting sites in order to understand the temporal and spatial biodiversity, and further enrich the information available for the specimens taken at the site. Another potential product we had not considered, but were encouraged to contribute data for, was a 3D panorama (our example can be seen here: http://www.3d-360.com/ ). These are achieved by shooting two panoramas of the same drawer at slightly different angles (i.e., positioning the drawer slightly to the left or right of center to capture different perspectives). Then independent, proprietary software is used to make the panorama visible in three dimensions, either using anaglyph glasses (red/cyan) or through other methods (e.g., cross-eyed viewing, etc.). Lastly, we used GigaPan to enhance the insect collection project for the NCSU ENT 502 graduate-level course, Insect Biodiversity and Evolution, by creating panoramas of the final collections submitted by several graduate students (http://gigapan.org/gigapans?order=most_popular&page=1&per_page=10&query=ent+502 ). The resulting panoramas effectively archived the students’ projects, either to remind them of their efforts or to guide future students making collections. We anticipate that the ease and adaptability of GigaPan will encourage even more creative applications of the technology to collection science.

### Workflow improvement

Project workflow varied little after initial setup and achieving the present results. Though we did not objectively and iteratively evaluate the process along the way, several observations were made based on user experience. During drawer imaging there is down time, even when using that time to prepare the next drawer (see Fig. 3). One option for taking advantage of this time might be the incorporation of a second system, so that two drawers could be imaged in a partly overlapping time frame. Employing additional people to capture the images would not be more efficient (unless more than two systems are used at once), though having one person image the panoramas and another person stitch them after each batch reduces time. Another step that could be streamlined is data transfer, which could be done wirelessly if such technologies were incorporated (for example a wireless memory cards for the camera; http://www.eye.fi/ ). Additionally, upgrading the entire system to use a Digital SLR would enable options for wireless file transfer, but at a greater total cost (in addition to the cost described below in *Advantages of GigaPan*). However, the small amount of time saved may not be economically worth it. An automated batch stitch and upload could be initiated overnight to save man hours, though software for doing so is not yet available. The only drawback would be the inability to identify and correct errors in the batch process until after time has been spent stitching the panoramas (as noted in Blagoderov et al. 2010).

There is a need to formulate objective ways to evaluate the quality of the panoramas, from aesthetics like resolution, exposure and clarity, to more scientific criteria such as the potential for identifications and the amount of data that can be observed in the drawers (e.g., from labels). Furthermore, errors, such as those encountered during capture and stitching (usually involving out of focus images and misaligned tiles, respectively), were usually identified before uploading, but some subtle ones still exist in panoramas present online. To rectify the situation it will be beneficial to identify the visual clarity of the panoramas and any persisting errors; crowd sourcing the panoramas to determine these quality metrics could help to expedite the process.

### General issues for mass imaging insect drawers

Imaging entire insect drawers with any system has its drawbacks. The following were identified by the authors early on, and reiterated in responses on a survey of the utility of the drawer panoramas for research (Hammond MS Thesis in prep).

Panoramas of pinned specimens tend to show only some angles of the insects; dorsal and some lateral aspects are usually visible, but ventral views are generally obscured. Limiting the observable amount of a specimen limits the power of these images for determining some species, especially ones where diagnostic characters are located in obscured areas. Lack of good image resolution and magnification associated with ordinary camera optics also hinders identification, especially for smaller specimens. Though higher magnification and resolution can be obtained for these panoramas, it usually involves taking more photos of each drawer (increasing time needed for the entire project) and purchasing special lenses that are often expensive and not always available for the system being used. Another result of a single overhead panorama is that larger specimens can hide labels, further reducing the amount of information available to viewers.

Collections are consistently being updated and curated, thus many panoramas derived from such a project will become out dated at different rates and not fully represent the current state of the collection. This occurs as specimens are added to and moved around the collection, rendering the drawer images inaccurate, especially in active sections of the collection. As such, we consider these panoramas to be “snapshots” of each drawer at the time of imaging, and we provide a date on each drawer after the initial capture to hopefully aid in future evaluations of the true level of change (or stasis) for each drawer. A method for labeling the level of curation on each drawer post-panorama (e.g., number of specimens added or taken from each drawer) would help to determine which drawer images need to be updated, though such a system is not yet fully formulated and could be complicated to implement and enforce.

### Advantages of GigaPan

Using GigaPan technology for drawer imaging is ideal in a number of ways. The entire system described here cost approximately $1,500 (US):

GigaPan Epic 100 (~$450)

Canon G11 (~$500)

lighting (~$500)

copy stand (~$100)

other accessories (~$50)

Upgrading to an Epic Pro (http://gigapan.org/cms/shop/epic-pro ), with a Digital SLR camera and its lenses, would increase the overall price by about $3,000. The moderate price of the system described here is financially accessible to many different collections: from small, personal collections to those with millions of specimens. The system is user-friendly, under normal circumstances after setup, initial data can be captured quickly and easily. The software is also easy to use and avenues for support are readily available through GigaPan.com. Furthermore, the ability to customize and adapt the system is highly advantageous because it does not limit the purchaser/user to particular hardware. For example, if a collection/laboratory already has an acceptable camera, it has the potential to be coupled with the system without the need to purchase a new one. Also, because the system was initially developed for work in the field, it could easily play a role in both “lab bench” research (as described here) and remote field work. Finally, the infrastructure to easily host, discuss, and annotate these immense panoramas is already present (i.e., GigaPan.com) and thus alleviates the need to invest in ways to locally disseminate the product (e.g., buying personal servers). All of these factors contribute to increased accessibility, a critical component for widespread adoption. The formation of a vast online community of collections, and the resulting communications, could be contingent on this ease of adoption.

### Limitations of GigaPan

**Figure 7.**
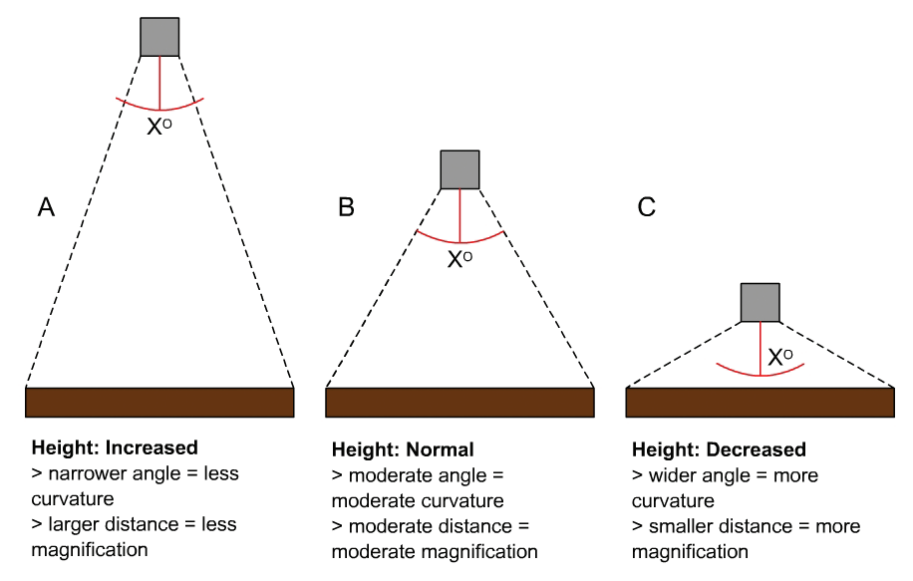
Illustration of the panning angle with the GigaPan robot at different heights. **A** higher than described **B** as described **C** lower than described.

The main difference between GigaPan and other image capturing/stitching systems is that the robot and camera are fixed and rotate around a central point. XY coordinate systems, on the other hand, pan across a fixed area and are shot in the same horizontal plane and at the same distance. Because GigaPan rotates around a point, there is always some curvature/distortion to the images (Fig. 6). The level of curvature is proportionate to the distance the unit is from the subject and the zoom (Fig. 7). Though the stitch software adjusts for these effects, measurements being made from the panoramas would not be accurate in portions of the image (see Results for distortion effects). Insects near the bottom of the drawer and their unit tray labels can be blocked by the leading edge of the unit tray, especially small trays with specimens close to the top edge. Additionally, while other drawer types (e.g., Cornell & California Academy styles) with similar dimensions should be easily accommodated using the methods described here, larger or custom drawers will need a greater distance between the insects and camera to keep the curvature to a minimum; this in turn would compromise the magnification of the images (without the use of special lenses). However, the curvature does allow for viewing vertically-oriented header labels in unit trays in the upper half of the panorama, more angled views of the insects (i.e., their sides), and specimen labels that are less hidden by the body of the insect (usually more hidden with a completely over-head camera, i.e. XY system). All of these results can actually be advantageous because they permit more information to be displayed in the panorama.

Other considerations are necessary for utilizing the system to its fullest. For an efficient workflow, an AC adapter should be integrated into the unit. The GigaPan robot normally runs on batteries that are quickly drained after several panoramas are shot. Rechargeable batteries last somewhat longer, but still need to be recharged and put back in the robot, which is time consuming; it also moves the robot, negating any saved coordinates and reducing overall efficiency. Integrating the adapter requires electrical knowledge, but can be done (Sargent et al. 2010). If the panoramas are going to be represented online an internet connection is necessary, preferably one with fast upload speed. This may be a limitation for some collections.

Annotating the panoramas on Gigapan.com is not as sophisticated as necessary for highlighting specific structures on an insect. Presently, only a rectangular snapshot can be made of an area in the panorama; more detailed description is then required to signify what the snapshot is showing. The development of better tools that could highlight specific structures would be beneficial for communicating information held within the panoramas.

### Future goals

The utility of a Digital SLR equipped with a macro lens should be tested for this system. We anticipate higher quality images with better resolution of smaller specimens using better optics, though we do not entirely know how larger cameras and lenses (and their intrinsic characteristics) will affect the process. This would require both an SLR camera and a larger GigaPan robot (i.e., Epic Pro). Adding a step for post-processing images in photo editing software (e.g., Adobe Photoshop) prior to stitching, in order to enhance the sharpness, color and exposure of the panoramas, may improve final image quality.

Ongoing efforts to database the collection and apply unique specimen barcodes could be integrated into the final product. Already several drawers online have barcoded specimens (for example http://gigapan.org/gigapans/69756 ), though most barcodes are obscured under other labels to save space. However, modifying drawers to have the barcodes visible could allow people browsing the collection to scan the codes on their computer screen to access relevant label data or populate a list of specimens needed for loan. The system could be useful for tracking specimens that move between drawers and link them to their placement in the most current panoramas.

Many future goals involve enriching these panoramas by integrating more layers of information. We anticipate adding more keywords to each panorama to enable more powerful searches. These would include lower taxonomic ranks (subfamilies, tribes, genera, and species) and perhaps general localities. There is a great benefit to linking other information to the panoramas. For instance, a snapshot of one species (or a series of specimens of one species) could be linked to the species’ detailed images found on Morphbank (http://www.morphbank.net/ ), biodiversity information from GBIF (http://www.gbif.org/ ), genetic sequence data from Genbank (http://www.ncbi.nlm.nih.gov/genbank/ ), and other sources like the Encyclopedia of Life (http://eol.org/ ), Tree of Life project (http://tolweb.org/tree/ ), and many others. Additionally, if structures can be more accurately annotated (see *Limitations of GigaPan*), they could be linked to data present in various anatomy and phenotypic ontologies (e.g., OBO Foundry; http://obofoundry.org/ ). The possibilities are vast, but would require some added infrastructure and resourcing to achieve these results.

Other research avenues for these panoramas should be assessed. Can specimens in the image be analyzed and identified using a computer algorithm and machine learning? Can text information be extracted from the visible labels? With correction techniques, can accurate measures and morphometric analyses be performed? Could we use these panoramas to profile the state and quality of each drawer in the collection (similar to criteria described in McGinley 1993 and Favret et al. 2007)? What can the panoramas tell us about color patterns within and between species? These are a few of the uses envisioned, though they are by no means the only possibilities.

## Conclusions

Overall, this project has generated excitement among entomologists and museum colleagues, which is encouraging for the future utility and adoption of this system. Many experts readily recognize the utility of drawer GigaPans, and the project has triggered several conversations about how to extend their outreach and research potential, as well as their ability to increase institutional awareness (both internally and externally). Though there are concerns about the full utility of these panoramas, especially the quality and nature of the images for identifying some insects, and their accuracy after the drawer contents go through curation, the low cost, ease of use, moderate speed, and online support make this technology a feasible system for imaging and sharing insect drawers from many settings.
